# The Effect of Tin on Microstructure and Properties of the Al-10 wt.% Si Alloy

**DOI:** 10.3390/ma15186350

**Published:** 2022-09-13

**Authors:** Janusz Kozana, Marcin Piękoś, Aldona Garbacz-Klempka, Małgorzata Perek-Nowak

**Affiliations:** 1 Faculty of Foundry Engineering, AGH University of Science and Technology, Reymonta 23 St., 30-059 Krakow, Poland; 2 Faculty of Non-Ferrous Metals, AGH University of Science and Technology, Mickiewicza 30, 30-059 Krakow, Poland

**Keywords:** cast technologies, cast alloys, material characterisation, aluminium, Al-Si alloys, Al-10 wt.% Si, tin, microstructure, mechanical properties, SEM-EDS

## Abstract

In this paper, the results from studies regarding near-eutectic Al-Si alloys with Sn as an alloying addition are presented. In most Al-Si alloys, tin is regarded as a contaminant; thus, its amount is limited to up to 0.3 wt.%. The few studies that can be found in the literature regarding the behaviour of tin in aluminium alloys suggest the beneficial effect of this element on selected properties. However, these results were obtained for hypereutectic Al-Si alloys or wrought aluminium alloys. In our studies, the influence of tin contents of up to 1.7 wt.% was determined on the AlSi10 alloy. Thermal analysis, measurements of the mechanical properties of the cast and heat-treated alloy, metallographic observations (light microscopy, scanning electron microscopy), and EDS (X-ray energy dispersive spectrometry) measurement allowed us to fully describe the effect of tin on the aluminium alloy. The results of the thermal analysis showed changes in the range of the α-Al solution crystallisation and the α+β eutectic through a decrease in the alloy’s solidification start point and eutectic solidification point. As a result, the elongation of the alloy was more than double in the AlSi10Sn1.7 alloy, with an A5 value of 8.1% and a tensile strength that was above 200 MPa.

## 1. Introduction

The exceptional popularity of aluminium alloys in the automotive [1,2,3,4,5], aerospace [6,7,8,9,10,11,12,13,14], space craft [9], and numerous other sectors is primarily due to their high strength-to-weight ratio, easy workability, and low cost.

The literature discusses detailed aspects of selecting aluminium alloys as lightweight materials as well as recent developments in the use of aluminium alloys in passenger cars and aviation for structural and engine components.

Both of the traditional techniques for manufacturing aluminium alloy components have been discussed, which include casting [4], metal-forming techniques [1,2], modern manufacturing methods [5], and component joining.

Through the introduction of alloying elements, aluminium alloys are characterised by very good mechanical properties, high plasticity, and satisfactory hardness [15,16]. They are also marked by excellent castability, low density, and good resistance to high-temperature and corrosive environments [17,18]. Aluminium alloys have been extensively researched with a view toward achieving high strength and durability. In order to improve the quality of castings and increase the areas of utilising aluminium alloys, the processes of selecting and optimising the chemical compositions [4,15,16] and the refining and modification processes [19,20,21,22,23] as well as selecting the casting technologies and heat-treatment parameters have been carried out [24,25,26]. These works discuss the dependence of the properties of aluminium alloys on their structures and describe the factors and methods that influence the development of modern aluminium alloys in order to improve their properties. Increasing the strength of these alloys allows for thinner components to be used; this results in reductions in weight, which in turn has a significant positive environmental impact. The introduction of alloying additives to the alloys of the Al-Si system provides special opportunities (as evidenced by the number of scientific works and experiments in this field). The small additions of elements, that act as modifiers, and greater additions of alloying elements, that were presented in refs. [19,27,28,29]) were investigated.

In ref. [30], an analysis of the phosphorus addition to the Al-Si alloy’s solidification process was carried out; the authors confirmed its significant influence on the nucleation process and silicon precipitation rate. In ref. [31], a titanium addition into aluminium with silicon alloys was experimentally checked. The authors confirmed that the Ti addition resulted in TiAlSi intermetallic phase precipitations. Thus, the hardness could be improved by increasing the Ti amount due to the increase in the volumetric share of the relatively hard intermetallic phases. Bismuth’s influence was evaluated as well—especially on the mechanical properties and wear resistance. In refs. [32,33], it was stated that it was possible to achieve a significant increase in tensile strength and wear resistance at around a 1 wt.% Bi content in the Al-Si alloy. Due to the complex modification mechanisms of the aluminium alloys, various alloying elements are very often examined for their impacts on the modification process. Such an approach was described in ref. [34] for an Sr-based modifier. The results of the complex experiments were published in which various chemical elements were jointly examined, as described in refs. [35,36]. In ref. [37], Mg’s influence on the microstructure and mechanical properties of Al-Si-Cu alloys [38], Al-Si-Mg-Cu alloys by thixo-cast [39], and Al-Si-Cu-Fe alloys for squeeze-casting applications was described. The authors determined the content of magnesium for the best performance. Copper also influenced the strength and hardness of the casting aluminium alloys [29,40].

Information about the exclusive influence of Mg, Ni, Cu, Ti, Mn, Be, Fe, and Sn can be found as well as the synergic effect of some of these. A similar approach was presented in ref. [41], where less common additions like Ag, La, and Ce were examined (again, singly and together). Other authors examined the high melting point elements in addition to aluminium-silicon alloys; an example can be found in ref. [42] where results for Cr, Mo, and V were presented. Review articles can be found where attempts were made to combine great knowledge in the area (as it was in ref. [43]) and where rare earth elements were analysed in addition to the previously mentioned additions. The influence of the chemical compositions of aluminium and silicon alloys on the processes at the border of foundry and plastic working (as described in ref. [44] for rolling) was also investigated.

The addition of tin has also been evaluated; however, it can be stated that, compared to other elements, tin remains insufficiently analysed. The sparse experiments in this area show that the addition of Sn into Al-Si alloys can perform many functions that allow for obtaining a balance between the strength and wear resistance of this alloy group [45,46,47,48].

During the analyses of the previous research, it can be stated that the addition of tin resulted in a considerable microstructure refinement, along with the even distribution of tin and silicon inside the α-Al alloy matrix of the Al-Si-Sn alloy. It is worth adding that such an effect was recorded for 25 wt.% Sn in the alloy; for such a content, a considerable increase in the wear resistance and reduction in the surface pressure were demonstrated. The addition of tin made these alloys less prone to abrasion than base aluminium–silicon alloys [49,50,51].

The tin effect on the hardness and tensile strength of the A390 alloy was researched as well [52], where it was stated that up to a 1% addition improved the mechanical properties much better than traditional heat-treatment procedures. Very interesting results were presented in [53,54,55], where a significant microstructure improvement was achieved with the addition of tin, which resulted in a considerable improvement in the mechanical properties. Even though alloys with Zn, Ni, and Bi additions were investigated in this case, the results nevertheless showed the enormous potential of Sn as an alloying element in aluminium alloys. The influence of Sn was tested not only for typical casting alloys but also for Al-Si alloy coatings that are obtained by laser techniques. The influence of tin on the microstructure and functional properties of coatings that are obtained in this manner has been described (for example, in [56]). The authors of [57] looked at Al-Si-Cu alloys and examined how the addition of Sn would affect their mechanical properties (including the addition of Ti). This confirmed the results that were presented in the previously cited sources. The authors of this publication focused their research on casting alloys; from the terms of the available results, the influence of tin on other properties in Al-Si casting alloys seems interesting.

The main aim of our research is to determine the method of interaction of tin additives that are introduced into aluminium-silicon alloys. 

## 2. Materials and Methods

This research aimed to analyse the melting technology and casting of aluminium alloys, focusing on an example of the AlSi10 alloy with tin additions that were within a range of 0.2–1.7% [58]. The scope of the work included the preparation of the following:

- charge materials;
- furnace with accessories;
- moulds for making castings;
- thermal analysis equipment.

As part of the research, melts that contained the planned chemical composition were conducted. The quality of the liquid alloy was determined in terms of the hydrogen content, and the refining process was carried out based on the reported needs. Individual alloys were poured into prepared moulds, and the temperature changes of the alloy during the solidification and cooling were registered.

The obtained castings were tested in terms of tensile strength, plasticity, and hardness. Metallographic observations of each alloy were also carried out using optical and scanning electron microscopy (SEM). The microstructure was observed in electron backscattered (BSE) contrast in order to better differentiate the formed phases according to their mean Z numbers. Additionally, the SEM equipment provided the opportunity to carry out an elemental analysis of the microregions with X-ray energy dispersive spectroscopy (EDS).

The additions were selected on the basis of their own experimental research so as to show the effects of Sn at different contents. The selection of the amounts of the additives was also preceded by an analysis of the phase diagrams [17,59,60,61,62,63].

### 2.1. Preparation of Alloys

The alloy-melting process was carried out in an electric induction furnace of a medium frequency with the use of a chamotte–graphite crucible. The crucible was cleaned of any metal residues and skim from the previous melting process.

Ingots of the AlSi7Mg0.3 alloy (AB-42100—acc. PN-EN 1676 EN, Alumetal S.A., Kęty, Poland) were used as the main charge. The alloy with a composition that was similar to theeutectic alloy (AlSi10) was obtained by adding crystalline silicon and master alloys (M-AlSi50—according to PN-EN 575, Alumetal S.A., Kęty, Poland).

Before charging the batch, the furnace was heated to remove any moisture from the crucible that could adversely affect the molten alloy. After the metal charge had melted, the bath was heated to a temperature of 750 °C, and the weighed amount of silicon was introduced as we waited for its dissolution. Due to the tendency of aluminium alloys to dissolve hydrogen, the quality of the metal bath was controlled, and gas refining (argon) was used. The metal-preparation process was carried out in such a way as to eliminate any gaseous impurities and solid inclusions that could have distorted the obtained results.

The well-prepared alloy was used to perform the planned research on the impact of variable tin additions to the AlSi10 alloy.

Ready alloys were cast into a metal mould in order to obtain castings for further research.

The chemical compositions of those alloys that were obtained according to the planned experiment were determined by an spark optical emission spectrometer (OES) (SPECTRO MAXx, Kleve, Germany). The chemical composition of the obtained alloys is presented in Table 1.

To assess the influence of the variable tin additions to the AlSi10 alloy, sample castings were made in metallic dies; these allowed for making castings in the shape of the sample for testing the mechanical properties. Before casting, the mould was heated to within a temperature range of 150–200 °C. The pouring temperature of the liquid melt was within a range of 730–750 °C. The set mould and melt temperatures were used for all of the scheduled melts.

Those castings that were made with the use of a metal mould were tested to determine their chemical composition, HBS hardness, UTS tensile strength, A elongation, microstructure, and machinability assessment.

### 2.2. Thermal-Derivative Analysis (TDA) Method

With the use of thermal-derivative analysis (TDA), the characteristic thermal effects that resulted from phase changes that occurred during the crystallisation of the tested AlSiSn alloys were recorded and determined. For the thermal analysis, a mantle K-type (NiCr-NiAl) thermoelement (diameter—0.5 mm) was used with a centrally located thermocouple at the top. The temperature measurements were made by a Keysight 34972A laboratory multimeter (Santa Rosa, CA, USA) equipped with a 16-channel Module 34902A Reed Multiplexer. The obtained data made it possible to conduct a thermal analysis of the tested AlSiSn alloys.

### 2.3. Thermodynamic Modelling

On the basis of the TDA curves and thermodynamic modelling, investigations of the phase analysis were also made. The thermodynamic analysis of the alloys was performed on the basis of the CaLPHaD method (CALcula-tion of PHAse Diagrams) with a packet Thermo-Calc ver. 3.1 for aluminium alloys TCAL2: TCS Al-based Alloy Database (TCAL).

### 2.4. Microstructure Analysis and Phase Analysis

Metallographic microstructure observations were carried out by optical microscopy (NIKON EclipseLV150, Tokyo, Japan) together with a camera and Nis-Elements (Melville, NY, USA ) image recording and analysis software ver. 3.22.15(Bulid 738). The SEM-EDS analyses were carried out with a Hitachi S-3400N electron microscope (Tokyo, Japan) with a tungsten filament as an electron source and an EDS detector by Thermo Noran. In addition, some SEM studies (including electron backscattered diffraction [EBSD] and an EDS element distribution map) were carried out with a Tescan Mira (Brno, Czech Republic) with an FEG electron column, SW version 1.1.3.0 buulid 4348, an EBSD Symetry S2 (produced by Oxford Instruments, Abingdon, UK), and an EDS Ultim Max (also produced by Oxford Instruments).

### 2.5. Mechanical Property Analysis

The UTS tensile strength and A elongation (number of tests—5) were determined by a LabTest ZD20 Labor Tech (V = 10 MPa/s), while the Brinell hardness HBS 2.5/62.5 measurements (number of tests—3) were made using an HPO-250 hardness tester (WPM, Labor Tech, Leipzig, Germany). The strength tests were carried out on samples without machining (as cast); this made it possible to evaluate selected properties of the analysed alloys without machining while at the same time allowing us to obtain results that determined the nature of the changes that occurred for the real castings that were machined only in certain important places.

Determining the mechanical properties of the analysed alloys was carried out in accordance with current standards while ensuring the accuracy of the measurements.

### 2.6. Machinability Tests

Machinability tests were carried out using the Keep-Bauer method [64,65]. This method consists of drilling holes of a predetermined diameter in a material (using a new drill for each test) at a constant feed force with drill bits. The determinant of machinability and, thus, wear resistance (in the cases of materials with such properties) is the change in the speed of the penetration of the drill over time. To assess the machinability of our alloys, a Ø5-mm drill was used (spindle speed—680 rpm; feed force—500 N). The machinability measurements were made in the area of the gripping part of each of the tensile strength test samples.

## 3. Results

### 3.1. Thermal-Derivative Analysis (TDA) Method

**Figure 1:**
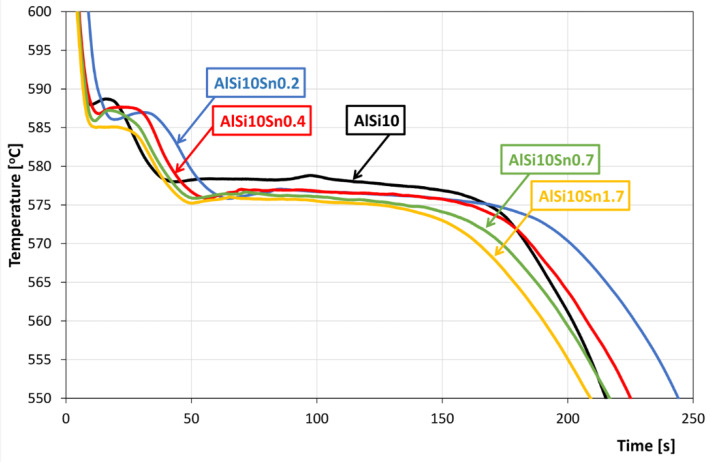
Cooling curves of AlSi10Sn alloys.

**Figure 2:**
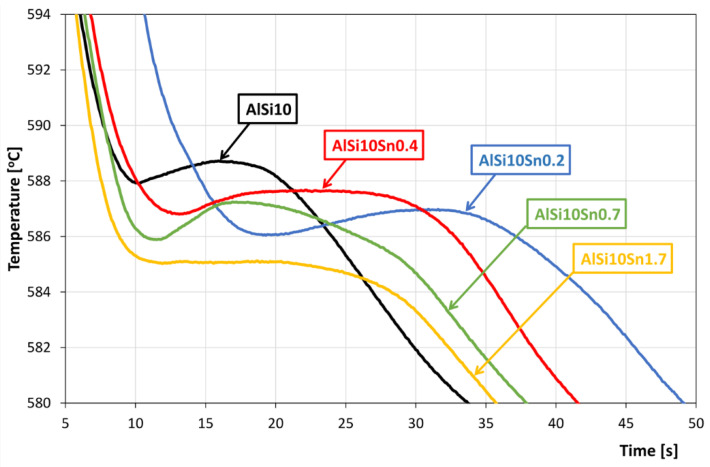
Part of AlSi10Sn alloy’s cooling curve diagram in terms of the onset of solidification of α-Al solid solution.

**Figure 3:**
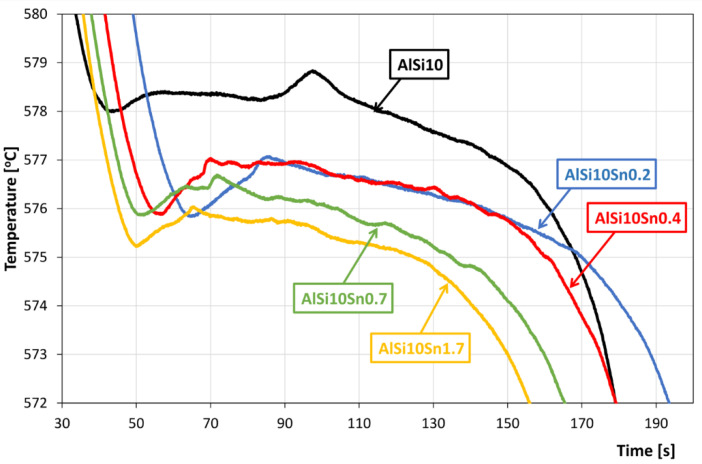
Part of AlSi10Sn alloy’s cooling curve diagram in terms of the onset of solidification of α + β eutectic.

The recording of the solidification and cooling of the analysed alloys allowed us to analyse the changes that occurred in the AlSi10 alloy with variable tin additions. An aggregate diagram of the alloy’s crystallisation is shown in Figure 1, and selected areas from the solid solution α-Al solidification and α + β eutectics are shown in Figure 2 and Figure 3.

**Figure 4:**
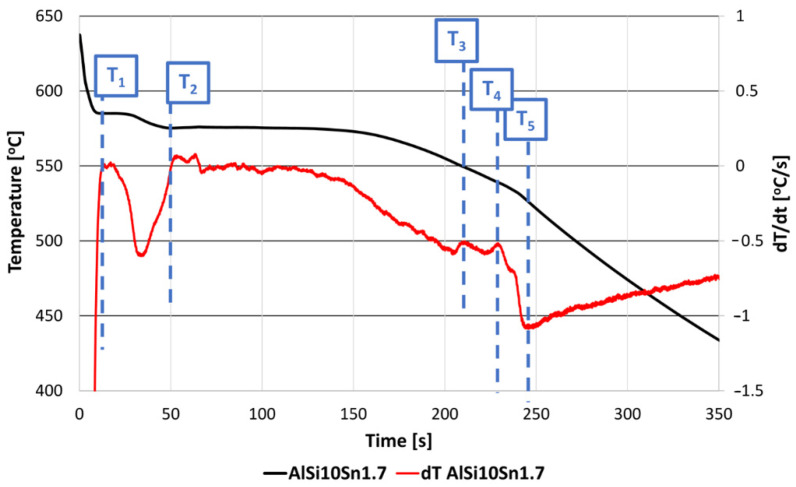
Characteristic temperatures of crystallisation were determined on the basis of TDA. T1—beginning of crystallisation (separation of primary α phase dendrites); T2—triple eutectic crystallisation (α + Al_9_Fe_2_Si + β); T3—triple eutectic crystallisation (α + Mg_2_Si + β); T4—triple eutectic crystallisation (α + Al_2_Cu + β); T5—end of crystallisation.

The thermal-derivative analysis made it possible to determine specific characteristic temperatures such as the beginning of the T1 crystallisation, the crystallisation of the T2 eutectic, the crystallisation of the T3 and T4 intermetallic phases, and the temperature at the end of the T5 crystallisation (Figure 4 for AlSi10Sn1.7). The obtained results are summarised in Table 2.

### 3.2. ThermoCalc Modelling

The modelling was carried out in the ThermoCalc program, which enabled a visualisation of the crystallisation process of the examined alloys. The modelling results were compared with the actual process that was recorded during the solidification of the tested alloys and the thermal derivative analysis performed in this way. Table 3 present the characteristic temperatures for the crystallisation processes of the selected alloys.

According to the results of the modelling of the crystallisation of the examined alloys at a temperature of 20 °C, there were two basic phases with the highest proportion in the alloy structure: the FCC_L12 phase (α phase) and the SI -DIAMOND_A4 phase (which corresponded to the crystal structure of the A4 diamond; in this case, it was pure silicon). In addition, there were phases with lower percentages: the AL9FE2SI2 phase, the AL3TI_D022 phase, and the AL2CU_C16 phase. These were multi-component phases that contained Fe, Ti, and Cu in the alloy. The Q_ALCUMGSI phase crystallised only in the AlSi alloy. The disclosed phase in the ThermoCalc modelling was the MG2SI_C1 phase, which changed its composition to Mg2Sn with a very low Si content when tin was added to the alloy. Tin was present in the alloy as a practically pure element in the form of the BCT_A5 phase. The content of the individual elements in the phases that occurred during the alloy crystallisation is presented in Table 4.

**Figure 5:**
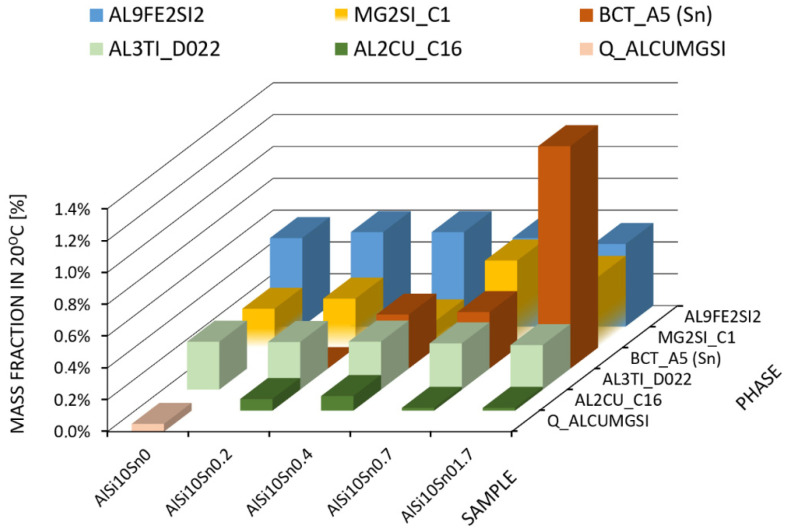
Graphical presentation of the share of selected phases that were obtained from TC modelling at ambient temperature (20 °C); FCC_L12 # 2 (Al) and DIAMOND_A4 (Si) phases not shown.

The results of the modelling of the alloys and the percentages of the individual phases that were obtained at an ambient temperature (20 °C) are presented in Table 5 (and graphically in Figure 5).

### 3.3. Microstructure Analysis and Phase Analysis

**Figure 6:**
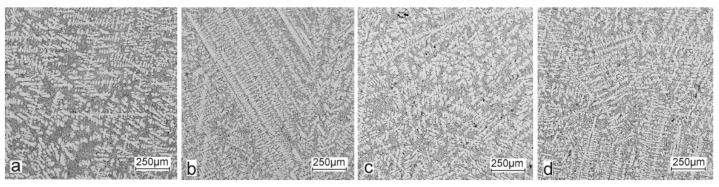
Microstructure of AlSi10 alloy with Sn addition—mag. 50×: (**a**) AlSi10; (**b**) AlSi10Sn0.2; (**c**) AlSi10Sn0.4; (**d**) AlSi10Sn0.7.

**Figure 7:**
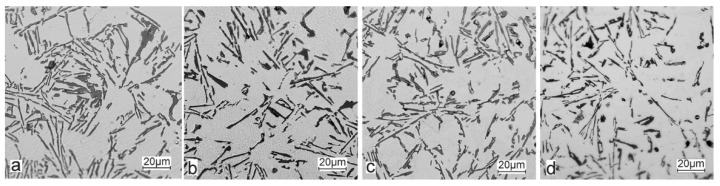
Microstructure of AlSi10 alloy with Sn addition—mag. 500×: (**a**) AlSi10; (**b**) AlSi10Sn0.2; (**c**) AlSi10Sn0.7; (**d**) AlSi10Sn1.7.

Selected results of the metallographic tests (OM) are presented in Figure 6 and Figure 7. The metallographic tests were carried out on the prepared samples to assess any changes in the microstructure (taking the individual precipitates into account).

**Figure 8:**
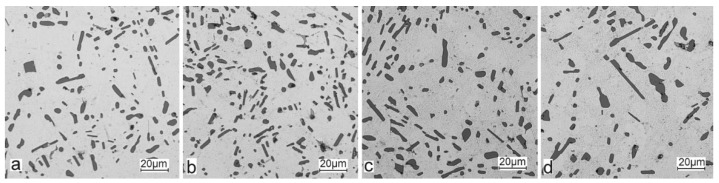
Microstructure of AlSi10 alloy with Sn addition after T6 treatment—mag. 500×: (**a**) AlSiSn0.2; (**b**) AlSi10Sn0.4; (**c**) AlSi10Sn0.7; (**d**) AlSi10Sn1.7.

The obtained castings from the analysed alloys were also subjected to T6 heat treatment (supersaturation—540 °C/6 h; aging—180 °C/12 h) (resistance muffle furnace—PEM-2). Selected results of the metallographic tests of the AlSi10Sn alloys after the T6 heat treatments are shown in Figure 8.

**Figure 9:**
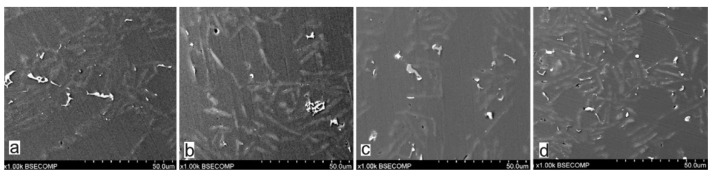
Microstructure of AlSi10 alloy with Sn addition (BSE—mag. 1000×): (**a**) AlSi10; (**b**) AlSi10Sn0.2; (**c**) AlSi10Sn0.4; (**d**) AlSi10Sn0.7.

**Figure 10:**
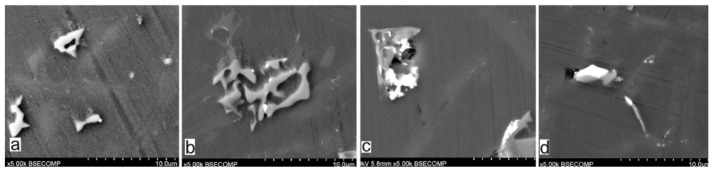
Microstructure of AlSi10 alloy with Sn addition (BSE—mag. 5000×): (**a**) AlSi10; (**b**) AlSi10Sn0.2; (**c**) AlSi10Sn0.4; (**d**) AlSi10Sn0.7.

**Figure 11:**
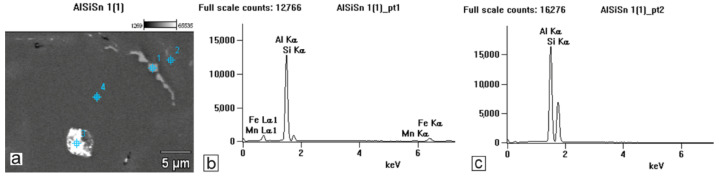
SEM-EDS analysis of microstructure and chemical composition of Si10Sn0.2 alloy: (**a**) image of microstructure with points of analysis; (**b**) plot of spectra in Area 1; (**c**) plot of spectra in Area 2.

**Figure 12:**
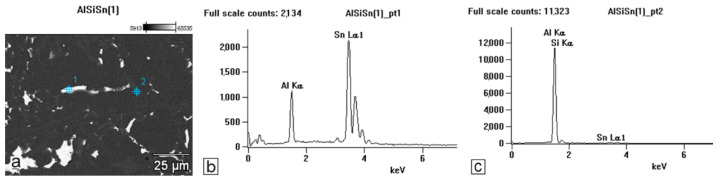
SEM-EDS analysis of microstructure and chemical composition of Si10Sn1.7 alloy: (**a**) image of microstructure with analysis points; (**b**) plot of spectra in Area 1; (**c**) plot of spectra in Area 2.

**Figure 13:**
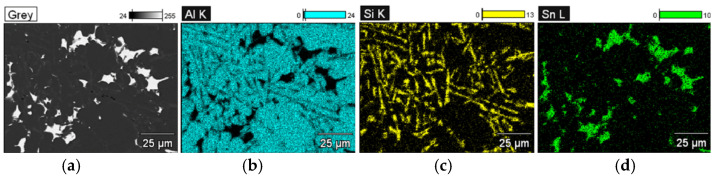
SEM-EDS analysis of microstructure and chemical composition of Si10Sn1.7 alloy (element distribution map—mag. 1000×): (**a**) image of microstructure; (**b**) Al; (**c**) Si; (**d**) Sn.

The studies that were carried out with the use of the scanning electron microscope allowed us to observe changes in the microstructure and identify the shapes of the tin precipitates that were used as an alloying addition to the AlSi10. These test results are presented in Figure 9, Figure 10, Figure 11, Figure 12 and Figure 13 and Table 6 and Table 7.

**Figure 14:**
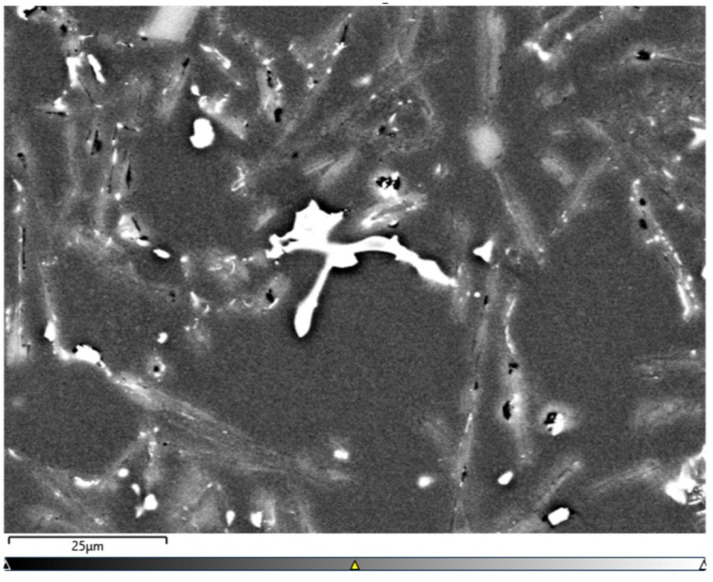
Microstructure of AlSi10Sn1.7 alloy with Sn addition (electron image).

**Figure 15:**
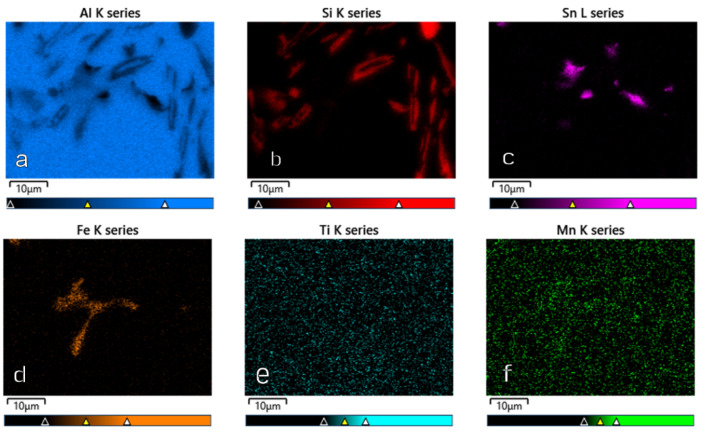
Element distribution maps for AlSi10Sn1.7: (**a**) Al; (**b**) Si; (**c**) Sn; (**d**) Fe; (**e**) Ti; (**f**) Mn.

**Figure 16:**
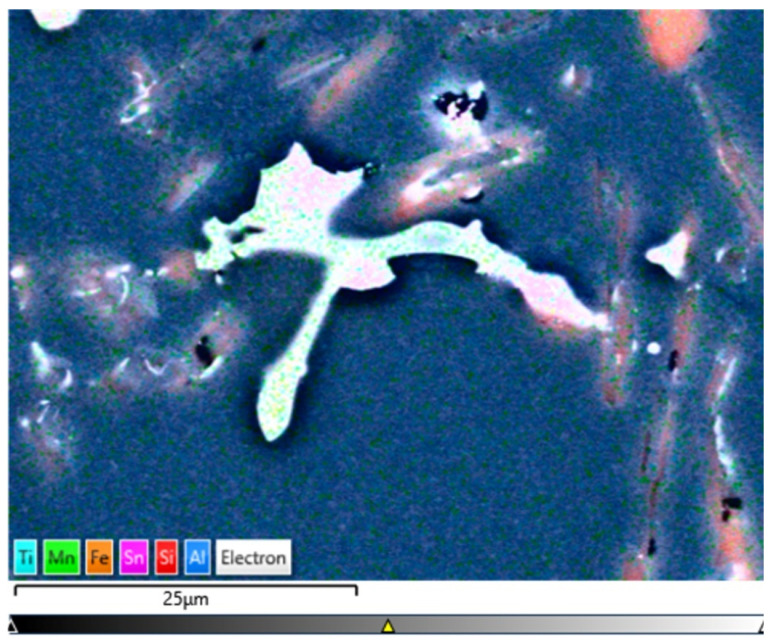
SEM-EDS layered image—AlSi10Sn1.7 alloy.

**Figure 17:**
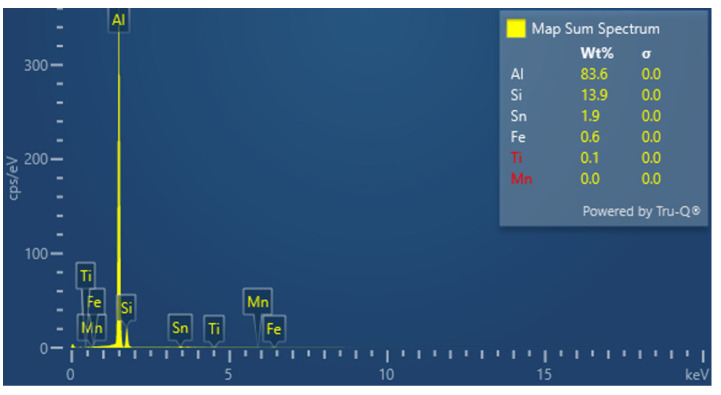
Spectrum of elements—AlSi10Sn1.7 alloy.

Figure 14, Figure 15, Figure 16 and Figure 17 show the microstructures of the AlSi10Sn1.7 alloy taken with a Tescan Mira scanning microscope, qualitative EDS analysis in the form of elemental spectra and elemental distribution maps.

**Figure 18:**
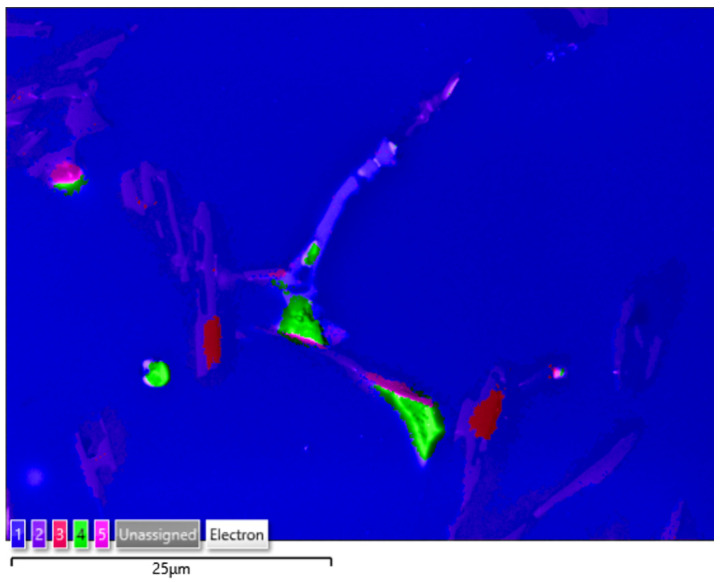
SEM-EDS image of AlSi10Sn1.7 alloy (1—Al; 2—AlSi; 3—Si; 4—SnAl; 5—AlSiSn).

**Figure 19:**
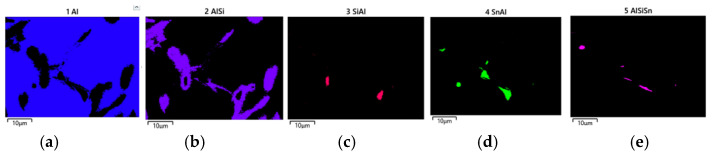
Phase distribution maps for AlSi10Sn1.7: (**a**) Al; (**b**) AlSi; (**c**) Si; (**d**) SnAl; (**e**) AlSiSn.

**Figure 20:**
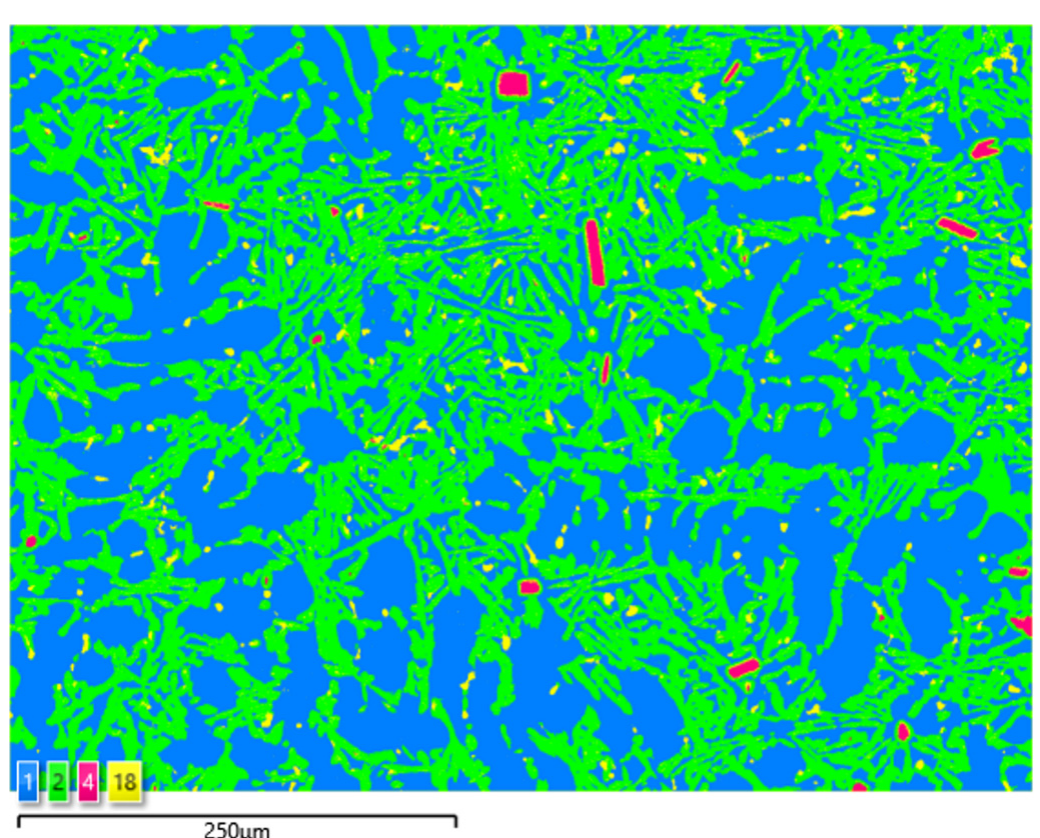
SEM-EDS image of AlSi10Sn1.7 alloy (1—Al; 2—AlSi; 4—SiAl; 18—AlSiSn).

**Figure 21:**
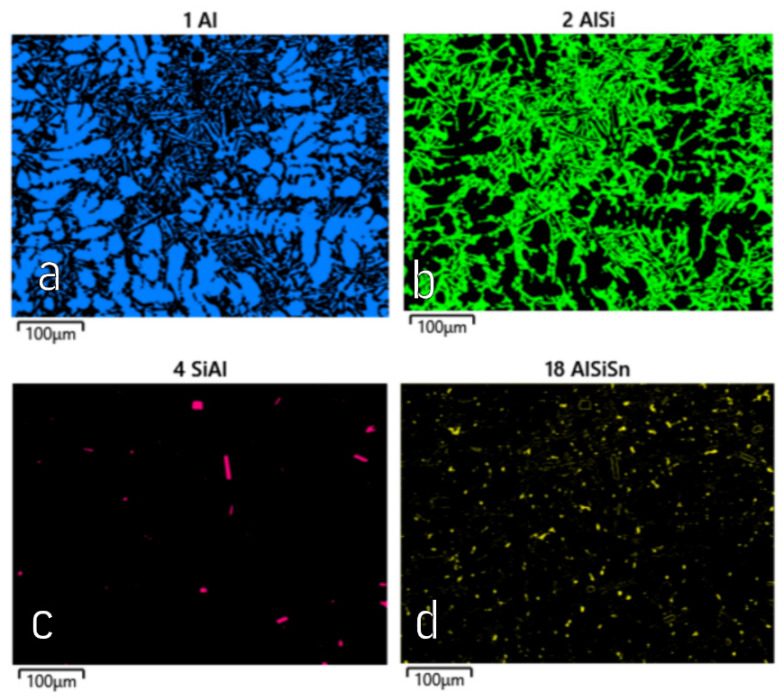
Phase distribution maps for AlSi10Sn1.7: (**a**) Al; (**b**) AlSi; (**c**) SiAl; (**d**) AlSiSn.

Based on the electron and EDS images, the phase image was obtained: Al (α); AlSi (α+β); Si (β); SnAl (Sn) (denoting pure tin); AlSiSn; SiAl (Si) (denoting pure silicon) (Figure 18, Figure 19, Figure 20 and Figure 21; Table 8 and Table 9).

### 3.4. EBSD Analysis

**Figure 22:**
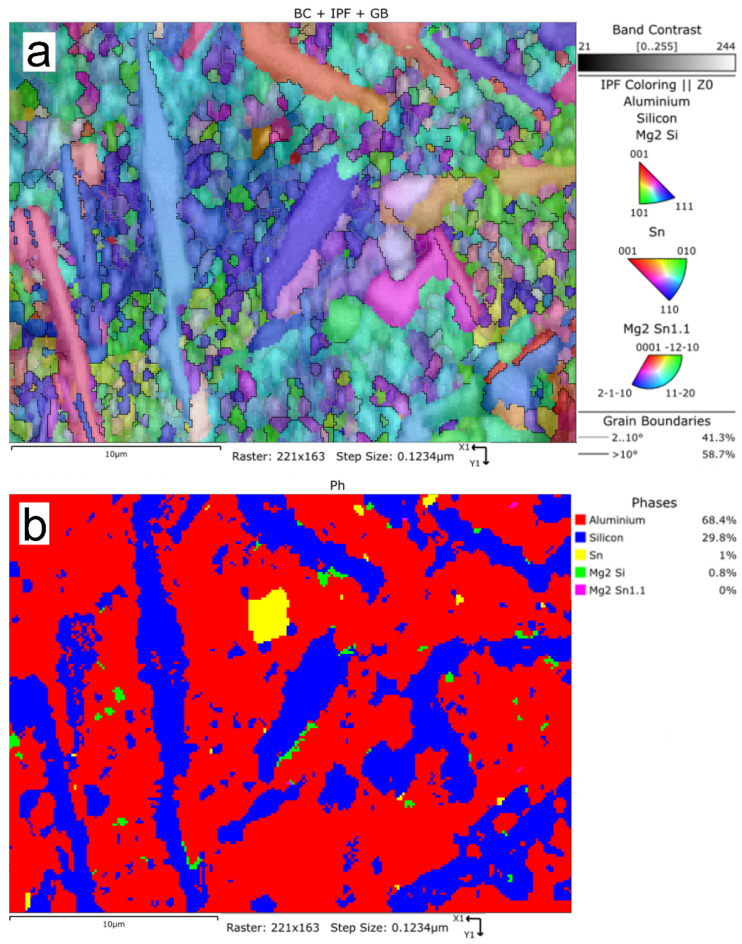
EBSD maps of AlSi10Sn1.7 alloy: (**a**) IPF map; (**b**) phase distribution map (red—Al; blue—Si; yellow—Sn; green—Mg2Si; pink—Mg2Sn1.1).

The EBSD analysis confirmed the presence of the following phases in the Al-Si-Sn alloy: aluminium, silicon, tin, Mg2Si, and Mg2Sn1.1 (Figure 22a,b). In Figure 22, the distribution of the crystallographic orientation of the mentioned phases is demonstrated in IPF colouring. The IPF colours were assigned according to the inserts in Figure 22a. Moreover, the phase distribution map with marked silicon crystals (blue) and tin precipitates (yellow) is presented in Figure 22b. Most of the Sn was located within the aluminium matrix as primary tin precipitates; only a small amount of Mg2Sn1.1 was found.

### 3.5. Mechanical Property Analysis

**Figure 23:**
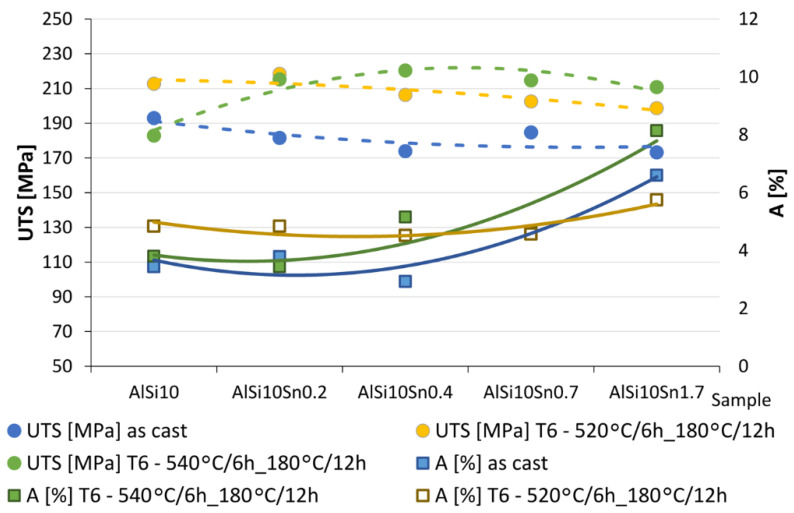
Results of the tests of tensile strength (UTS—dashed line) and elongation (A—solid) for AlSi10 alloys with variable tin additions. Blue line—as cast; green line—T6—540 °C/6 h_180 °C/12 h; yellow line—T6—520 °C/6 h_180 °C/12 h.

Strength tests were carried out on the samples without machining; this allowed for an evaluation of selected properties of the analysed alloys without machining and, at the same time, allowed for obtaining the results that determined the nature of the changes that took place for the real castings (which, for obvious reasons, were machined only in specific places that were important). The results of the obtained tests are presented in Table 10 and Figure 23.

**Figure 24:**
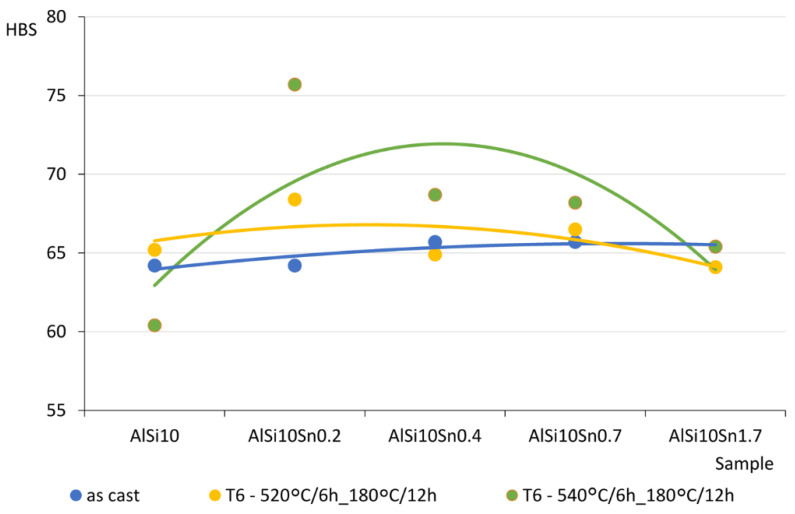
HB hardness test results for AlSi10 alloys with variable tin additions. Blue line—as cast; green line—T6—540 °C/6 h_180 °C/12 h; yellow line—T6—520 °C/6 h_180 °C/12 h.

The obtained and analysed results indicated a slight decrease in the tensile strength of the analysed AlSi10 alloys that had increased tin content. The plasticity of the tested alloys (determined by elongation parameter A) improved slightly. The results of the hardness tests of the AlSi alloys with variable tin additions are presented in Table 10 and Figure 24.

The obtained Brinell hardness (HB) values of the analysed alloys did not show any significant changes from the impact of the variable tin additions (up to 1.7%).

### 3.6. Machinability Tests

**Figure 25:**
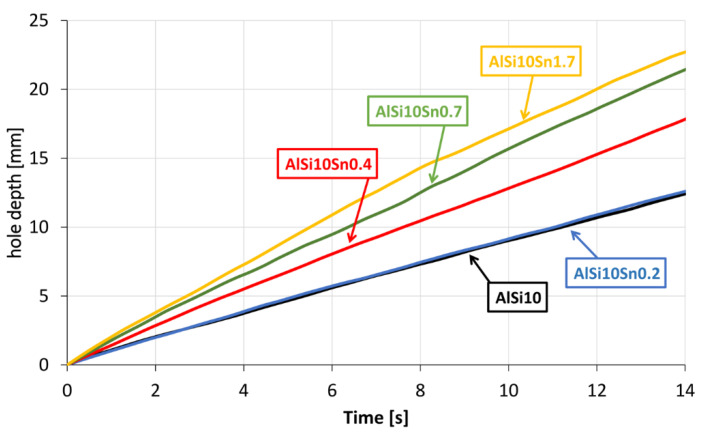
Results of machinability tests of AlSi alloys with variable Sn additions.

The next stage of the research included assessing any changes in the machinability of the analysed alloys. The results of these tests are presented in Figure 25 in the form of a graph.

## 4. Discussion

### 4.1. Thermal-Derivative Analysis (TDA) Method

An analysis of the obtained results showed the clear influence of the variable tin additions to the analysed AlSi10 alloy. With increases in the proportion of tin, the crystallisation temperature of the α-Al solid solution decreased, and the supercooling effect in this area disappeared. This is related to the formation of the nuclei of the crystallisation, the growth of the dendrites of the solid solution, and the thermal effect of the process. Changes could also be observed in the area of the α+β eutectic solidification.

The solidification point of the T2 eutectic decreased from a value of 578.0 °C (for the AlSi10 starting alloy) to a value of 575.2 °C (for the AlSi10Sn1.7 alloy). For all of the analysed alloys, a recalescence effect could be observed, i.e., supercooling in the initial area of the eutectic crystallisation. The range of the T1–T5 crystallisation also expanded.

### 4.2. Thermodynamic Modelling

According to the TC modelling, the addition of tin to the AlSi10 alloy caused its appearance in the process of the crystallisation of the second liquid (“LIQUID_2”), which was difficult to detect in the case of the TDA.

**Figure 26:**
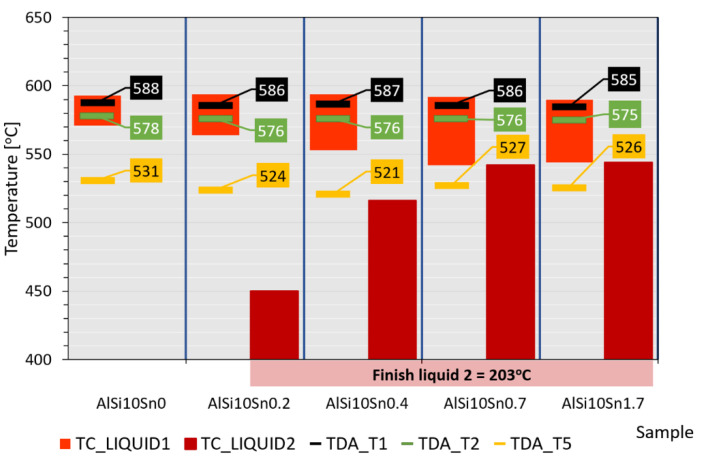
Schematic representation of crystallisation process by TC modelling (characteristic points of TDA marked).

Figure 26 shows a schematic presentation of the crystallisation process of the examined alloys. The range of the occurrence of LIQUID_1 liquids increased from 22 °C (for the AlSi10 alloy) to 46 °C (for AlSi10Sn1.7); also, the introduced addition of tin caused the temperature of the beginning of the LIQUID_2 liquid to change from 450 °C to 544 °C. The end of the LIQUID_2 crystallisation was 203 °C for those alloys with Sn additions. Additionally, the characteristic temperatures that were recorded during the performed thermal-derivative analysis, the beginnings of the T1 crystallisation and T2 eutectic crystallisation, and the end of the T5 crystallisation were plotted.

The obtained results of the TC modelling and TDA were convergent in terms of the crystallisation start temperature and eutectic crystallisation temperature—the differences were maximums of 7 °C and 2 °C, respectively. This convergence was mainly due to the relatively high proportion of aluminium and silicon. The remaining elements that remained in smaller proportions and occurred as alloying additives (Mg, Ti, and Sn) or were present as impurities (Fe, Mn, Cu, and Zn) caused thermal effects during the crystallisation; however, they did not directly correlate with each other.

### 4.3. Microstructure Analysis and Phase Analysis

A microscopic observation clearly showed the presence of eutectic silicon in the α-Al solid solution.

The metallographic tests of the analysed AlSi10 alloys with variable tin additions showed the dendritic nature of the structure of the α-Al solid solution and the precipitations of eutectic silicon in the interdendritic spaces; these are especially visible in the photos that were taken at a 50× magnification.

For those alloys without heat treatment, the eutectic showed an unmodified state, with the silicon in the forms of plates/needles that were arranged in a disordered manner (sometimes forming the shape of a eutectic grain). One can also notice the precipitation of larger silicon crystals—the structure of which indicates that these were the primary silicon. With this group of alloys—the near-eutectic group, and with the actual solidification conditions in the mould, which were significantly different from the equilibrium conditions, it is not excluded. The spheroidisation of the eutectic silicon precipitates can be observed in the images of the microstructure of the samples after T6 heat treatment. This phenomenon is well-known for AlSi alloys and leads to improvements in certain properties of such castings.

The tests that were carried out with the use of the scanning electron microscope allowed us to observe changes in the microstructure and identify the form of the tin that was used as an alloying addition for AlSi10.

The SEM-EDS analyses are shown in Figure 9 and Figure 12 as well as in the corresponding Table 6 and Table 7. Tin is visible in separate irregularly shaped separations (Figure 11a and Figure 12a) and confirmed in the spectra (Figure 11c and Figure 12b) and the tables (Table 6 and Table 7).

Based on the electron images, colour maps of the distribution of the elements in the microstructure were also made (Figure 13, Figure 14, Figure 15, Figure 16, Figure 17, Figure 18, Figure 19, Figure 20 and Figure 21). Based on selected mapping images, the proportion of the individual alloying elements was determined (Table 8 and Table 9).

The tin that was introduced into the AlSi10 alloy was visible in the form of very fine microdispersive precipitates in the matrix and as separate precipitates of aluminium and tin with variable amounts of other elements, such as magnesium, iron, manganese, and silicon.

The applied EDS element distribution map revealed the form of the tin that occurred in the AlSi10 alloy.

The tin-containing phases featured shapes that were somewhat aligned with the interdendritic spaces. In the area of the precipitates, slightly rounded depressions could be observed; this corresponded to the filling of the spaces around the dendrite arms. This may have been due to the accumulation of free tin or Sn-Al eutectic precipitates in the interdendritic spaces or around those complex intermetallic phases that were associated with the presence of iron and manganese in the alloy. The introduced tin accumulated in the spaces of these complex crystallised AlSiFeMn phases.

### 4.4. Mechanical Property Analysis

The obtained and analysed results indicated slight decreases in the tensile strengths of the analysed AlSi10 alloys with increased contents of tin. The plasticity of the tested alloys (determined by elongation parameter A) improved slightly, and the obtained Brinell hardness (HB) values of the analysed alloys did not show any significant changes from the impact of the variable tin additions (up to 1.7%).

### 4.5. Machinability Tests

The obtained curves that showed the speed of the penetration of the drill bit when drilling the Ø5-mm hole with a constant feed force showed the favourable variable effect of the tin addition. As the Sn alloy addition to the AlSi10 alloy increased, the drilling speed also increased. It can be assumed that the occurring tin-related precipitates caused chip breakage and thus reduced the cutting resistance.

## 5. Conclusions

Tests were carried out for a near-eutectic AlSi10 alloy with variable tin additions that ranged from 0.2 to 1.7 wt.%. The produced alloys were cast into a metal mould.

The conducted research allowed us to draw the following conclusions:

- A TDA of the obtained alloys showed changes in the onset of the solidification as well as in the solidification period of the eutectic. The addition of tin to the analysed AlSi10 alloy lowered the solidification point of the α-Al solid solution with the simultaneous disappearance of recalescence in this area. The eutectic solidification point also decreased by about 3 °C for the AlSi10Sn1.7 alloy.
- Thermodynamic modelling using CAL-PHAD with Thermo-Calc software provided an overview of the crystallisation process of AlSi10 alloy with tin additives with special attention to the dual liquid phase (Liquid_1, Liquid_2). Tin occurs as separate separations of the BCT_A5 phase and in a phase with magnesium identified by Thermo-Calc as Mg_2_Si_C1 (Mg_2_Sn).
- The metallographic tests that were carried out by light microscopy showed the classic structure of the microstructures of the analysed AlSi alloys. The solid solution of the silicon in the aluminium α-Al adopted a dendritic structure, and the eutectics that formed during the crystallisation were located in the interdendritic spaces. Eutectic silicon adopted the structure of the plates/needles in a random arrangement, and the T6 heat treatment caused the spheroidisation of the eutectic silicon precipitates.
- The studies, which were carried out using a scanning electron microscope, confirmed the thermodynamic modelling of CAL-PHAD (Thermo-Calc). The EBSD analysis confirmed the presence of the following phases in the Al-Si-Sn alloy: aluminium, silicon, tin, Mg_2_Si, and Mg_2_Sn. Tin was mainly visible in separate irregularly shaped particles and in small secretions in the form of the Mg_2_Sn phase.
- The obtained results of the mechanical properties did not show significant changes in the tensile strength and hardness of the analysed alloys. As a result of introducing tin in amounts of up to 1.7%, the plastic properties (determined by elongation parameter A) improved—reaching a value of more than 6% for the AlSi10Sn1.7 alloy. The addition of tin significantly improves the machinability of the alloy, thus causing an increase in the speed of drilling a hole of a certain diameter at a constant feed force.

## Figures and Tables

**Table 1:** Chemical composition of AlSi10 alloys with tin addition.

No.	Si	Fe	Cu	Mn	Mg	Zn	Ti	Sn	Al
	(wt.%)								
0	10.4	0.15	0.01	0.01	0.17	0.01	0.11	0	Bal.
1	10.1	0.16	0.04	0.01	0.09	0.02	0.11	0.22	Bal.
2	10.1	0.16	0.05	0.01	0.05	0.02	0.11	0.46	Bal.
3	10.2	0.15	0.01	0.01	0.16	0.02	0.11	0.74	Bal.
4	10.1	0.14	0.01	0.01	0.13	0.02	0.10	1.71	Bal.

**Table 2:** Characteristic temperatures of crystallisation were determined on the basis of TDA.

Sample	T_1_	T_2_	T_3_	T_4_	T_5_
	[°C]				
AlSi10Sn0	587.9	578.0	564.4	545.8	531.4
AlSi10Sn0.2	586.1	575.8	564.2	540.2	523.5
AlSi10Sn0.4	586.8	575.9	566.9	536.2	520.7
AlSi10Sn0.7	585.9	575.9	550.8	539.3	525.6
AlSi10Sn1.7	585.1	575.2	549.0	539.0	526.0

**Table 3:** Example of TC modelling for AlSi10 and AlSi10Sn0.4 alloys.

Sample	Temperature [°C]/Mass Fraction in 20 °C [%]								
	T_1_	T_2_	T_2_	T_3_	T_4_	T_5_	T_6_	T_7_	T_8_
AlSi10Sn0									
LIQUID	593/100	576/80	573/17	571/0	-	-	-	-	-
FCC_L12#2 (Al)	593/~								20/88.6
DIAMOND_A4 (Si)	-	576/~							20/10.2
AL9FE2SI2	-	-	573/~						20/0.6
MG2SI_C1	-	-	-	-	-	405/~			20/0.2
AL3TI_D022	-	-	-	-	-	-	360/~		20/0.3
Q_ALCUMGSI	-	-	-	-	-	-	-	190/~	20/0.05
AlSi10Sn0.4									
LIQUID_1	594/100	576/78.1	572/19.8	553/0		-	-	-	-
LIQUID_2	-	-	-	-	516/~	358/0.4	346/0.4	203/0.0	-
FCC_L12#2 (Al)	594/~								20/88.5
DIAMOND_A4 (Si)	-	576/~							20/10.0
AL9FE2SI2	-	-	572/~						20/0.6
MG2SI_C1	-	-	-	-	-	346/~			20/0.2
AL3TI_D022	-	-	-	-	-	-	358/~		20/0.3
BCT_A5 (Sn)	-	-	-	-	-	-	-	203/~	20/0.3
AL2CU_C16	-	-	-	-	-	-	-	169/~	20/0.1

**Table 4:** Content of individual elements in phases that occurred during crystallisation of examined AlSiSn alloys.

Phase	Mass Percent of Element in Phase [%]						
	Al	Si	Sn	Fe	Mg	Ti	Cu
FCC_L12#2 (Al)	100.0	-	-	-	-	-	-
DIAMOND_A4 (Si)	-	100.0	-	-	-	-	-
AL9FE2SI2	64.11	8.92	-	26.97	-	-	-
MG2SI_C1	-	36.60	-	-	63.36	-	-
MG2SI_C1 (Mg2Sn)	-	0.23	70.49	-	29.27	-	-
AL3TI_D022	62.86	-	-	-	-	37.14	
Q_ALCUMGSI	21.59	26.96	-	-	31.11	-	20.34
BCT_A5 (Sn)	0.003	-	99.997	-	-	-	-
AL2CU_C16	45.92	0.0001	0.0008	-	-	-	54.08

**Table 5:** Results of modelling of alloys (mass fraction at 20 °C (%)).

Phase	FCC_L12#2 (Al)	DIAMOND_A4 (Si)	AL9FE2SI2	MG2SI_C1	AL3TI_D022	Q_ALCUMGSI	BCT_A5 (Sn)	AL2CU_C16
Sample	[%]							
AlSi10Sn0	88.6	10.2	0.56	0.24	0.30	0.05	-	-
AlSi10Sn0.2	88.7	10.0	0.59	0.31	0.30	-	0.003	0.07
AlSi10Sn0.4	88.5	10.0	0.59	0.17	0.30	-	0.340	0.09
AlSi10Sn0.7	88.1	10.1	0.56	0.55	0.29	-	0.355	0.02
AlSi10Sn01.7	87.2	10.2	0.52	0.44	0.28	-	1.397	0.02

**Table 6:** Chemical composition of AlSi10Sn0.2 alloy in micro area from Figure 11.

Sample	Mg	Al	Si	Mn	Fe	Sn
	Weight %					
AlSi10Sn0.2_pt1	-	68.33	7.30	1.51	22.86	-
AlSi10Sn0.2_pt2	-	56.07	43.93	-	-	-
AlSi10Sn0.2_pt3	1.60	20.46	13.86	-	2.55	61.53
AlSi10Sn0.2_pt4	-	98.58	1.42	-	-	-

**Table 7:** Chemical composition of AlSi10Sn1.7 alloy in micro area from Figure 12.

Sample	Al	Si	Sn
	Weight %		
AlSi10Sn1.7_pt1	10.08	-	89.92
AlSi10Sn1.7_pt2	90.45	6.11	3.44

**Table 8:** Phases in AlSI10Sn1.7 alloy in micro area from Figure 18.

Layer Number	Phase	Fraction (%)	Pixel Count
1	Al (α)	81.4	160,069
2	AlSi (α + β)	15.6	30,686
3	Si (β)	0.5	920
4	SnAl (Sn)	1.2	2377
5	AlSiSn	0.4	760
6	Unassigned pixels	0.9	1796

**Table 9:** Phases in AlSI10Sn1.7 alloy in micro area from Figure 20.

Layer Number	Phase	Fraction (%)	Pixel Count
1	Al (α)	52.7	1,657,705
2	AlSi (α + β)	43.8	1,377,174
4	SiAl (Si)	0.5	15,144
18	AlSiSn (Sn)	3.0	95,705

**Table 10:** Results of the tests of tensile strength (UTS) and elongation (A) for AlSi10 alloys with variable tin additions—as cast and T6 (solution heat-treated and fully artificially aged).

	As Cast			T6—540 °C/6 h 180 °C/12 h			T6—520 °C/6 h 180 °C/12 h		
Sample	UTS [MPa]	A [%]	HBS	UTS [MPa]	A [%]	HBS	UTS [MPa]	A [%]	HBS
AlSi10	193.0	3.4	64.2	182.9	3.8	60.4	212.7	4.8	65.2
AlSi10Sn0.2	181.5	3.8	64.2	215.3	3.4	75.7	218.5	4.8	68.4
AlSi10Sn0.4	173.9	2.9	65.7	220.4	5.2	68.7	206.4	4.5	64.9
AlSi10Sn0.7	184.7	4.7	65.7	214.6	4.6	68.2	202.5	4.6	66.5
AlSi10Sn1.7	173.2	6.6	65.4	210.8	8.1	65,4	198.7	5.8	64.1

## Data Availability

The data that support the findings of this study are available from the corresponding authors (J.K., M.P., A.G-K, M.P-N.) upon reasonable request.
